# Feasibility of a Social Network-Based Physical Activity Intervention Targeting Vocational School Students: A Pilot Study

**DOI:** 10.3390/ijerph19159474

**Published:** 2022-08-02

**Authors:** Liane Günther, Sarah Schleberger, Claudia R. Pischke

**Affiliations:** Institute of Medical Sociology, Centre for Health and Society, Medical Faculty, Heinrich-Heine-University Düsseldorf, Moorenstraße 5, 40225 Düsseldorf, Germany; sarah.schleberger@med.uni-duesseldorf.de (S.S.); claudiaruth.pischke@med.uni-duesseldorf.de (C.R.P.)

**Keywords:** physical activity, social media, vocational students, pilot study, Facebook

## Abstract

Globally, four out of five adolescents do not meet the recommendations for physical activity (PA). Moving large segments of young adults from inactivity to activity is essential to reach the global target of a 15% relative reduction in inactivity by 2030 worldwide. This study aimed to examine the feasibility of a social network-based PA intervention (WALK2gether) in vocational school students. Fourteen students from one vocational school in the city of Duesseldorf were instructed to walk ten thousand steps per day over six weeks. Applied behavior change techniques were self-monitoring of steps and social comparison via a pedometer app and a Facebook group. Indicators of feasibility were documented. The intervention was minimally resource intensive, with a total of 92 h spent by the research staff. The recruitment rate was 19.2% and loss-to-follow up 28.6%. Our data revealed no significant change in the target behavior PA from baseline to follow-up. The target population did not interact in the Facebook group, while a moderate use of the pedometer app was noted. Although the results ought to be interpreted with caution due to the small sample size, the findings suggest that the WALK2gether intervention was partially feasible, but not appropriate for the target group.

## 1. Introduction

### 1.1. Background

It is well known that regular physical activity (PA) during childhood and adolescence is beneficial for cardiorespiratory fitness, weight status, muscular strength, and bone health and that these preventive effects of regular PA are likely to accumulate until adulthood [1]. Unfortunately, four out of five adolescents aged 11–17 years did not meet the World Health Organization (WHO) recommendation of at least 60 min of moderate to vigorous physical activity (MVPA) per day and muscle strengthening exercises three days per week in 2016 [2]. Should public health strategies not be successful in moving large segments of adolescents from inactivity to activity, it is unlikely that the global target of a 15% relative reduction in inactivity by 2030 will still be met [3]. There are multiple settings for PA promotion that young adults can be targeted in Germany, one of them is the school setting. In Germany, three units (one unit = a 45 min lesson) of physical education are mandatory [4], but recently published survey data revealed that participation in physical education is the lowest among adolescents aged 14–17 years, with an average of 2.03 units spent in physical education and 23% in extracurricular sports activities [5]. Social media is defined as “network community communication channels” [6] (p. 38) and seems to be a promising tool for PA promotion in adolescents [7]. From 2012 to 2018, the daily use of social media doubled among 13–17-year old teenagers (from 34% to 70%) [8]. Characterized by participatory features, social media is used for social interaction and processes of social influence may encourage behavioral changes, as shown in a systematic review by Hosseinpour and Terlutter [9]. They found that competition and social sharing of PA performances with familiar users are the third most effective techniques for promoting PA. Zhang et al. (2016) discovered that social comparison in online social networks had an even more pronounced effect on PA-levels than social support [10]. Although it is known that students at vocational schools are at high risk for insufficient PA [11], they continue to be underrepresented in PA intervention studies. Given the relevance of social media in this age group, a Web 2.0-based PA program for PA promotion seems promising in this population. However, before conducting a full-fledged resource-intensive controlled intervention study, it is advisable to examine the feasibility of the intervention and the implementation processes involved [12].

According to the framework of Thabane et al. [12], the following characteristics of feasibility ought to be covered in a feasibility study piloting an intervention:

- Processes for successful implementation of the intervention, which are documented and rated,
- Resources necessary for the implementation of the intervention, which are tracked and quantified,
- Challenges during the development and implementation of the intervention, which are noted,
- Resources and challenges faced during data processing, which are recorded,
- Use and acceptability of the employed intervention (in our case the Web 2.0 platforms), as well as reasons for non-use, which are assessed.

### 1.2. Objectives

Hence, the overall aim of this pilot study was to examine the feasibility of a social network-based PA intervention that targets young adults in vocational schools based on the indicators outlined in the framework by Thabane et al. [12] (see above). Secondly, the magnitude of potential effects was determined, including an estimation of the possible magnitude of intervention effects on the primary outcome PA and an estimation of the possible magnitude of intervention effects on the secondary outcomes body mass index (BMI), subjective health status, quality of life (QOL), and exercise motives.

## 2. Materials and Methods

### 2.1. Participants and Setting

Participants were students of a vocational school in the city of Duesseldorf between 16 and 27 years old. They were eligible for study enrolment, if they met the following inclusion criteria: (a) had an internet-enabled smartphone, (b) had an active Facebook account or were willing to set up one for the intervention, (c) were willing to set up an account for the pedometer app, Pacer, (d) had no medical conditions or absolute contraindications prohibiting PA or exercise and (e) were German speaking.

In order to reach vocational school students, eight out of twelve vocational schools in the city of Dusseldorf were selected and a community-based-sampling strategy was applied. First, the school administration of each school was contacted via email to inform them about the study and to initiate the cooperation. Teachers from schools that were willing to participate offered access to pre-selected classes for the study staff to provide information and for recruitment.

**Figure 1:**
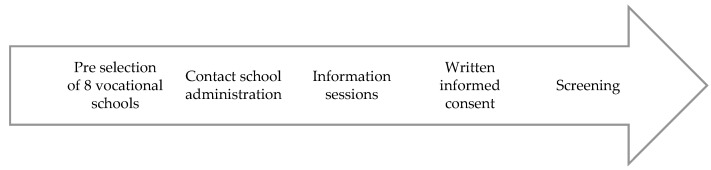
Five stages of the community-based sampling.

Interested participants were handed a written informed consent and asked to return it to their vocational school teacher before baseline assessment. After screening these participants for eligibility with a paper-pencil questionnaire that was completed immediately before baseline measurement (T0), participants were formally enrolled in the study and asked to set up their Facebook and Pacer accounts and join the private Facebook group created by the study staff. Then, participants of each class were prompted to form equal teams consisting of 3–8 individuals and to choose one team captain. This formation process was intended to encourage students to walk in self-selected teams of close friends or classmates. Team captains had organizational tasks, e.g., to set up their group in the pedometer app Pacer and invite their teammates. After the team building process was completed, the study staff offered time for questions and troubleshooting regarding the use of Facebook and Pacer. Additionally, a tutorial on how to use the most relevant features in Pacer and Facebook was provided in the joint Facebook group. Figure 1 illustrates the five different stages of the community-based sampling of vocational school students.

### 2.2. Intervention

The intervention WALK2gether is adapted based on the Active Team intervention by Maher et al. [13] and designed as a resource-friendly standalone digital intervention aimed at increasing PA. Self-monitoring and social comparison were the behavior change techniques (BCTs) that were used to motivate the students to walk 10,000 steps per day over a period of 6 weeks [14]. In the design process, we deliberately chose to use an online social network used by the general public and did not develop an app for the study because we wanted to pilot the use of already existing platforms.

A private group administered by the study coordinator in Facebook helped organize the study-related issues (e.g., termination of measurements, reminder to return accelerometers) and communicate with participants. Over the six-week period, the study coordinator provided information four times to reinforce the steps goal (e.g., a YouTube clip with tips for daily walking routines, also see Table 1).

In addition, the pedometer app Pacer was utilized to assist participants in monitoring their daily steps. Pacer has a built-in group feature to reinforce sharing individual PA and to foster social comparisons. Individually logged steps were automatically displayed at the leader board and ranked from high to low. This kind of friendly rivalry between classmates was intended to encourage participants in achieving 10,000 steps every day. WALK2gether took place between November 2021 and January 2022.

### 2.3. Outcomes

The main outcome of this study was feasibility which was evaluated following the framework of Thabane et al. [12]. Processes involved during the development and implementation of the intervention and study, resources required, and the management of challenges were documented. Quantitatively assessed parameters were captured at the baseline (T0) and at follow-up (T1) after completion of the six-week intervention to estimate the magnitude of the potential intervention effects. Data on PA, health outcomes, and participant feedback were collected in person by trained study staff at the Heinrich-Heine-University Duesseldorf (see Table 2).

Habitual PA was tracked with an accelerometer (wGT3X-BT, ActiGraph, Pensacola, FL, USA). Participants were instructed to wear the device on their non-dominant wrist during the day for one week and acceleration was recorded along three axes with a sample rate of 30 Hz. Valid wear-times were filtered with the algorithm of Choi et al. [15] and comprised at least four days with a minimum of ten hours (600 min) wear-time. By using the cut-off points for MVPA, according to Freedson et al. [16], the data were analyzed within a 10-s epoch, using the ActiLife 6.13.4 software. Participants were asked to simultaneously keep an activity diary to account for non-wear time. In addition, the German version of the IPAQ-SF was used to subjectively assess PA [17]. MVPA, walking, and sedentary time were calculated, using the cut-off points recommended by the IPAQ Research Committee [18]. The IPAQ has a good test-retest reliability (r = 0.80) [19], but a low criterion validity (r = 0.30) [19]. Weight (kg) and height (cm) were assessed by the study staff at the baseline and follow-up to calculate the BMI (kg/m²), using a scale (Seca 899) and a stadiometer (Seca213). Participants had to rate their subjective general health on a scale ranging from one (very bad) to five (very good) [20]. The WHOQOL-BREF is a 26-item questionnaire that was used to investigate QOL, in general, and the four dimensions physiological health, psychological health, social relationships, and environment [21,22]. The degree of internal consistency for the subscales has been previously shown to vary between α = 0.57 and α = 0.88 [23]. Fourteen motives for exercise participation were assessed with the German version of the Exercise Motivations Inventory (EMI-2) [24].

Structured feedback regarding the Web 2.0 platforms was gathered via a self-generated questionnaire. Additionally, a German version of the MARS scale (MARS-G) was employed to examine the quality of Pacer regarding objective dimensions, such as engagement, functionality, esthetics and information quality, as well as subjective app quality and perceived impact. The MARS-G has good internal consistency for all subscales (ω = 0.82, 95% CI 0.76–0.86) and good validity in all dimensions compared to the original version (r = 0.93 to 0.98) [25].

All questionnaires were converted to online surveys, using the program Lime Survey 5.2.8+220103, and could be accessed via a URL at both assessment points on two iPads.

### 2.4. Statistical Methods

As there is no need for a formal sample size calculation in a pilot study for a phase III trial according to Thabane et al. [12], sample size was not calculated. Sociodemographic sample characteristics were analyzed descriptively. The magnitude of the potential treatment effects was estimated with a *t*-test (IBM SPSS 25), performing intra-group comparisons regarding PA and secondary health outcomes. Results are shown as means ± standard deviations or percentages. Potential effects are displayed in standardized mean differences, including confidence intervals.

### 2.5. Feasibility Criteria

The study is considered feasible if at least 20 participants are deemed eligible for inclusion and decide to participate. Furthermore, a loss to follow-up rate of 10% was considered as likely. Assessments were not supposed to take more than 70 min per person at T0 and 85 min at T1. We expected many questions during the screening for medical contraindications for PA and missing data for questions about social status. The total time expense for the study ought to be manageable by one full-time employee. As the intervention was digital, and therefore time- and location-independent, we expected an attendance rate of 100% regarding the Facebook viewings. Furthermore, we considered the intervention as feasible if the estimation of potential intervention effects revealed a trend towards an improvement in all outcome parameters (i.e., PA, BMI, subjective health status, QOL and exercise motives) or at least maintenance at a high level. We assumed that Pacer would, on average, be used daily and Facebook to a lesser extent.

### 2.6. Ethical Aspects

This study was approved by the Ethics Committee of the Medical Faculty of the Heinrich-Heine-University Duesseldorf, Germany, on 4 June 2020 (Study-No.: 2020-860) and was conducted in accordance with the ethical principles of the World Medical Association’s Declaration of Helsinki [26]. Participants 18 years or older gave their own consent and for participants under the age of 18 years, at least one of their parents provided informed written consent.

## 3. Results

### 3.1. Recruitment

The first attempt to initiate a cooperation with eight vocational schools in Duesseldorf took place in June 2020. Due to the start of the COVID-19 pandemic, which led to recurring school closures, no school was at first willing to participate in the study. A second attempt was made in April 2021, still under COVID-19 restrictions, which finally resulted in a cooperation with one of the eight local vocational schools. In order to achieve certain representativeness, students of two different educational tracks at this school were invited to participate in the study. While two classes were pursuing an educational track focused on economics in the 11th and 12th grade, a third class was enrolled in an educational track that focused on health and social affairs in the 11th grade. All three classes were striving for an entrance qualification for enrolment at universities of applied sciences.

**Figure 2:**
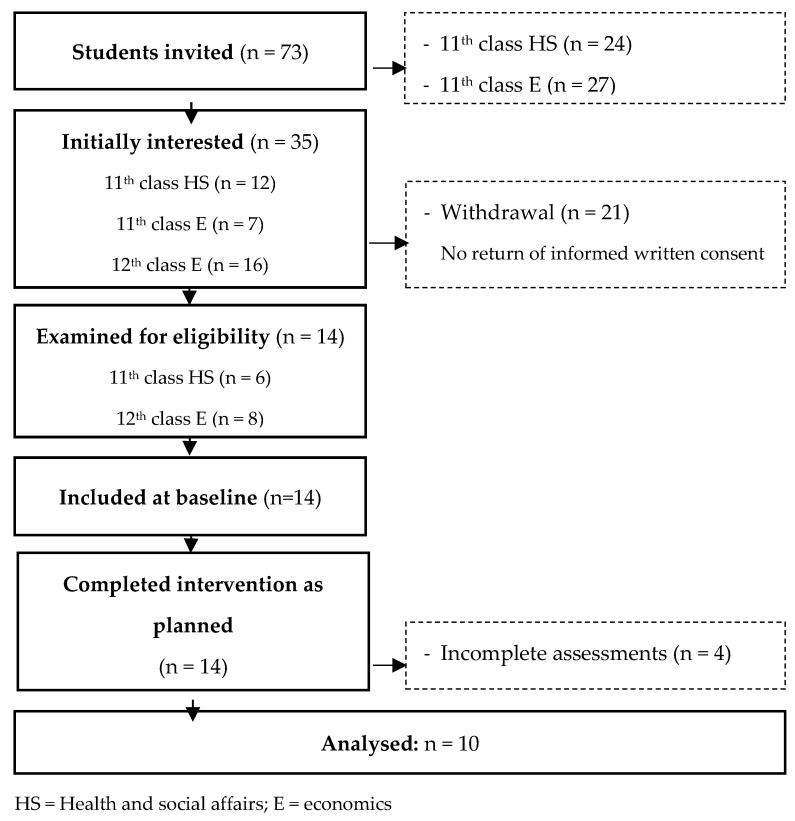
Recruitment rate and participant flow.

Recruitment took place at the end of October until the beginning of November 2021, baseline assessments were carried out subsequently, and follow-up assessment started mid-January 2022, immediately after the completion of the six-week intervention. Detailed information on recruitment rates and participant flow throughout the study is provided in Figure 2.

### 3.2. Participants

Fourteen vocational school students from two classes participated in the study, seven were female and eleven had a migration background. All participants had an intermediate graduation and rated their subjective social status (SSS) as moderate (5–6) on the MacArthur scale for adolescents (range including minimum of one and maximum of ten [27]) (see Table 3).

### 3.3. Feasibility

In total, 35 (48.0%) of the invited vocational school students were interested in participating in the study and 21 (28.8%) withdrew from participation by not returning completed consent forms to their teachers. Of the 14 (19.2%) vocational school students who were willing to participate, all met the eligibility criteria. Due to illness, four of them dropped out at the follow-up assessments. Facebook posts were, on average, watched by six participants, with no likes and just one comment was provided. During T0 and T1, no questions from the participants regarding the assessments were raised. However, during data analysis, frequently missing responses were found in the screening questionnaire with regard to items on household income. Nine participants did not give an estimate and seven participants did not assign household income to any given category.

Overall, study staff invested 92 h in the WALK2gether study and EUR 1535 costs emerged for the required measurement equipment (two iPads, two stadiometers, and two scales). Participants, on average, needed 34 min to carry out the baseline assessments and 28 min for the follow-up assessments, plus another 10 min per participant for the set-up of Pacer and to enter the Facebook group. The reception of all intervention materials provided by study staff took approximately 22 min per participant (see Table 4).

Most challenges were faced during recruitment and assessment. Due to the ongoing COVID-19 pandemic, we had to adjust to the constantly changing hygiene rules, recurring school closures, cancellations of lessons and illness (i.e., COVID-19 infections of students and/or teachers). This resulted in a shortened intervention period from eight to six weeks, as well as limited access to the target group via community-based sampling. In total, only three classes from one school could be recruited to the study. Furthermore, the assessments had to be relocated from the study site to vocational schools, so that two or more researchers could assess all participants of one class simultaneously during class or free time between classes in the gym or a recreation room. Due to the scarcity of time, paper-and pencil versions of the questionnaires were used instead of the online surveys.

Furthermore, participants were neither familiar with Facebook nor with Pacer and during the installation and we found that, depending on the latest software update, versions and functionalities of the two platforms differed. We, therefore, provided two tutorials that demonstrated the use of the most important app functions. Pacer seemed to have a technical bug with several participants reporting their steps not being transferred, resulting in zero steps being displayed in the individual and team statistics.

Overall, communication with participants during the intervention period via Facebook was difficult and teachers, therefore, often served as mediators. Vocational school students seemed to be less compliant when they were approached outside the school setting as reflected in the low response, as well as long return periods or non-use of study materials (e.g., accelerometers).

### 3.4. Estimation of Potential Effects

The results regarding PA at T0 and T1 were only reported for subjective PA (based on the questionnaire data derived from the IPAQ-SF), because analysis of the accelerometer data revealed that five participants wore the accelerometer at T0 and T1, but none of them reached the minimum of four valid wear time days. These results are discrepant with the PA diaries, which were completed by eight participants, indicating that at least four participants should have had valid accelerometer data for a minimum of four days. Subjectively rated PA (MVPA and walking time) and time spent in the sedentary state slightly increased from T0 to T1. BMI and subjective health status (predominantly rated as “good”) remained constant over the intervention period. Regarding QOL, different trends were observed for the different domains. In the physical health domain, QOL decreased from baseline to follow-up, whereas in the other domains, QOL increased and was most pronounced for the domain environment. Challenge, enjoyment and ill-health-avoidance were predominantly listed as exercise motives at T1 and all other motives were mentioned less or almost equally often after the six weeks of intervention. None of these results reached statistical significance (see Table 5).

### 3.5. Participants’ Feedback

Four participants reported that they used Pacer at least several times per week and some of them even daily (n = 4), mostly to monitor their individual step statistics (n = 8) and also to view the team ranking (n = 4). Six students stated that they would continue to use Pacer after the completion of the study. The overall quality of Pacer was rated as fair (3.3 ± 0.5). Its functionality was rated best (3.6 ± 0.7), followed by esthetics (3.2 ± 0.7). Lowest ratings were noted for the subscale information (3.1 ± 0.6) and engagement (3.1 ± 0.6). Subjective app quality was evaluated as moderate (2.8 ± 0.6).

Feedback pertaining to the utilization of Facebook was rather critical. Half of the respondents stated that they used Facebook less than once per week and three said they never used it. Reasons were lack of time and meaning (n = 2) and forgetting about it (n = 1). Only three participants reported that they used the private Facebook group for organizational matters. Furthermore, use of Facebook was declined by eight participants and seven participants stated that they would have liked to use another social media channel, with Instagram being the most popular choice (n = 5), followed by WhatsApp (n = 3).

## 4. Discussion

In the present study, we found that a social network-based PA intervention that targeted young vocational school students was partially feasible. We were not able to recruit the intended sample of 20 vocational school students and our loss to follow-up was nearly three times higher as expected. Both can certainly be explained by the fact that the study was carried out during the fourth wave of the COVID-19 pandemic in Germany. At that time, in-person contacts were restricted to a minimum; therefore, visits of the vocational schools for recruitment and assessment were severely restricted. Additionally, the incidence rates had reached a peak and schools, in particular, were affected by the illness of students, resulting in their absence at the follow-up assessment. The study turned out to be as resource friendly as intended. Both the amount of work, as well as the study material and associated costs, were manageable. Most assessments seemed to be appropriate for the target population. They took, on average, less than half of the scheduled time and, apart from the incomplete questionnaire data regarding household income, no questionnaire data were missing. However, irrespective of the pandemic, WALK2gether did not have the desired effect, because neither an improvement in PA nor a positive change in the other health outcomes from T0 to T1 could be observed. Nevertheless, subjective health status, BMI, and general QOL were maintained at relatively high initial levels. Participants’ feedback revealed a poor acceptance of Facebook, while Pacer was moderately used by the target group.

The aim of WALK2gether was to promote PA in a sample of vocational school students with an intervention strategy that was minimally supportive and related to Web 2.0 use behavior of adolescents. However, our results suggest that this was not appropriate for the target population. Students of both classes showed limited engagement with the intervention and study, whenever they were not supervised by either their teacher or study staff. This became evident by the high non-use rates of the study materials, such as accelerometers, activity diaries, and Facebook and led to the assumption that the target group needs a very structured regimen in a supportive environment to adopt behavioral changes in PA. A pilot study from Finland indicated higher engagement and acceptability of an environmental approach to promote PA in the school setting, but, similar to our results, reported that their optional website was barely used by the target group [28]. However, this is contrasted by the findings of Kuipers and colleagues [29], who reported that autonomous motivation is associated with increased MVPA in vocational school students and sustainability in behavior changes. They call for interventions that foster competence paired with autonomy, conducive to self-beliefs in individual success and confirm that peer relations are supportive for autonomous motivation.

The selection of adequate social media platforms for the target population is controversial. In a focus group study, vocational school students rated Facebook and text messaging as feasible methods for PA interventions [30]. These findings can neither be confirmed by our results, nor by the study of Saez et al. [31], who found that a Facebook challenge group was barely used by overweight, socially disadvantaged adolescents in a nutritional program.

The harmful consequences of social media use should always be carefully weighed against the benefits. Social media can cause stress, anxiety and depressive symptoms among adolescents due to envy and comparison and these effects are more pronounced in a mentally challenging crisis, such as the COVID-19 pandemic [32].

One limitation of this pilot study is the very small sample size that does not allow for preliminary estimations of the intervention effects and restricts the generalizability of the described findings. Another key limitation is the selection bias. Due to the COVID-19 pandemic, recruitment was performed at only one school and in three pre-selected classes. Furthermore, migration background and prevailing cultural and social norms regarding PA, which may influence the behavior, were not assessed in this small pilot study, but will be assessed in the full trial that will be conducted in the future [33]. One strength was the use of the framework of Thabane et al. [12] and the feasibility criteria specified a-priori.

## 5. Conclusions

Despite several limitations, this small pilot study provided important information regarding the needs and behavior of the target group of vocational school students and implementation processes involved when delivering a social media-based intervention. The results will inform the development and implementation of future social media-based PA interventions on a larger scale. Based on our results, we conclude that an optimization of WALK2gether is necessary and the following key points should be considered during the adaptation process:

- Creating a supportive environment, including integration of the program in the daily setting of the target group, e.g., school increasing autonomous motivation via peer relations and social support.
- Providing a social media add-on, including incorporation of an existing social media platform that is frequently and easily used by the target population during their leisure time.
- Addition of BCTs, including complementing the intervention with other effective BCTs, such as feedback and goal setting.

## Figures and Tables

**Table 1:** Digital intervention materials.

What	Content	Intervention Week
Tutorials app usage	Screen recording on how to use *Facebook* and *Pacer*	1
Post team ranking	Screenshot of team leader board of each group	2
Spotify podcast	“Quarks Daily” episode with scientific background information about the PA recommendation of 10,000 steps	3
YouTube clip	Video with tips on how to implement steady walking routines in everyday life	5

**Table 2:** Overview of feasibility outcomes and assessments.

	Parameter	Measurement Tool	Measurement Time
**Process**	Recruitment rate	Documentation	Implementation
	Withdrawal		Implementation
	Drop outs/completion rate		Implementation
	Attendance on Facebook		Implementation
	Adequacy eligibility criteria		Implementation
	Adequacy assessments		Implementation
**Ressources**	Study staff	Documentation	Entire study
	Time expenditure		Entire study
	Material		Entire study
	Costs		Entire study
**Management**	Challenges	Documentation	Entire study
	Solution strategies		Entire study
**Preliminary effects**	Objective PA	Accelerometer, activity diary	T0, T1
	Subjective PA	IPAQ-SF	T0, T1
	BMI	Stadiometer + Scale	T0, T1
	Subjective health	Questionnaire	T0, T1
	Quality of Life	WHOQOL-BREF	T0, T1
	Exercise motives	EMI-2	T0, T1
**Feedback**	Usage and acceptability	Questionnaire	T1
	Facebook and Pacer		
	Quality Pacer	MARS-G	T1

**Table 3:** Sociodemographic characteristics of the sample at baseline.

	Total (n = 14)	Male (n = 7)	Female (n = 7)
**Age, years**	17.4 ± 1.2	18 ± 1.5	17.1 ± 0.7
**Body mass index, kg/m^2^**	23.5 ± 4.8	21.3 ± 5.4	25.4 ± 2.3
**Subjective social status**	5.7 ± 1.7	5.3 ± 2.4	6 ± 0.8
**Migration background**	78.6	28.6	50
**Graduation (O-levels)**	100	50	50
**MVPA/day < 60 min**	71.4	21.4	50

Data are shown as mean ± standard deviation or percentage, MVPA = moderate to vigorous physical activity derived from IPAQ-SF.

**Table 4:** Expended time and staff resources.

What		Time (h)	Study Staff (n)
**Recruitment**		14.5	1–2
**Assessments**	Organization	33	3
	Implementation	15	2–3
**Intervention**	Design	14	1–2
	Implementation	1	1
**Data management**		15	1

**Table 5:** Estimation of treatment effects.

	T0	T1	SMD (95%CI)	*p*
**Subjective PA**				
MVPA (min/day)	42.1 ± 55.6	59.7 ± 52.9	−17.6 (−67.1, 31.8)	0.440
MVPA walking (min/day)	112.6 ± 113.4	125.1 ± 92.6	−12.5 (−122.4, 97.4)	0.803
Sedentary time (min/day) *	360 ± 49	381.4 ± 94.4	−21.4 (−117.2, 74.3)	0.604
**Subjective health**	2.1 ± 0.9	2.2 ± 1.1	0.1 (−0.5, 0.7)	0.726
**BMI**	23.5 ± 4.8	23.5 ± 4.5	0 (−0.4, 0.5)	0.887
**QOL (4–20)**				
General	14.4 ± 3.1	14.4 ± 3	0 (−1.8, 1.8)	1.0
Physical health	15.7 ± 2.8	15.3 ± 2.2	0.4 (−1.5, 2.2)	0.663
Psychological health	13.7 ± 3.7	14 ± 3.5	−0.2 (−3, 2.5)	0.846
Social relations	14.7 ± 2.2	14.9 ± 2.9	−0.3 (−2.8, 2.2)	0.814
Environment	14.7 ± 3.5	15.9 ± 2.8	−1.2 (−4.5, 2.1)	0.427
**Exercise motives (0–5)**				
Affiliation	2.2 ± 0.9	1.9 ± 1.5	0.3 (−1, 1.6)	0.582
Appearance	3.6 ± 1	3.3 ± 1	0.3 (−0.4, 0.9)	0.401
Challenge	3.3 ± 1.1	3.7 ± 1.1	−0.4 (−1.4, 0.6)	0.395
Competition	2.2 ± 1.5	2.2 ± 1.5	−0.1 (−1.5, 1.4)	0.940
Enjoyment	3 ± 1.3	3.4 ± 0.8	−0.4 (−1.4, 0.6)	0.404
Ill-health-avoidance	3.5 ± 1.2	4.2 ± 0.5	−0.7 (−1.7, 0.2)	0.109
Nimbleness	3.3 ± 1.1	3.4 ± 0.8	−0.1 (−0.9, 0.7)	0.787
Positive health	4.3 ± 0.8	4.2 ± 0.5	0.1 (−0.6, 0.8)	0.714
Revitalization	3.6 ± 1.1	3.8 ± 0.8	−0.2 (−1, 0.6)	0.604
Social pressure	0.7 ± 1.0	0.5 ± 0.8	0.1 (−0.6, 0.9)	0.701
Social recognition	1.4 ± 1.2	1.4 ± 1.0	−0.1 (−1, 0.8)	0.856
Strength and endurance	3.9 ± 1.5	3.6 ± 1	0.3 (−1, 1.6)	0.608
Stress nanagement	2.7 ± 1.2	2.8 ± 1.3	−0.1 (−0.7, 0.6)	0.806
Weight nanagement	2.2 ± 1.7	2.4 ± 1.7	−0.2 (−1.3, 1)	0.727

Data are based on n = 10 and are shown as mean ± standard deviation or percentage, SMD = standardized mean differences, CI = confidence intervals, PA = physical activity, MVPA = moderate to vigorous physical activity, BMI = body mass index, QOL = quality of life. * Data based on n = 7, n = 3 answered “I do not know”.

## Data Availability

The raw and statistical data of this study are available upon request from the corresponding author. The data are not publicly available due to reasons of privacy.

## Abbreviations

BCT	behaviour change technique
BMI	body mass index
MVPA	moderate to vigorous physical activity
PA	physical activity
QOL	quality of life
SSS	subjective social status
WHO	World Health Organization
